# Heat Conduction in a Functionally Graded Plate Subjected to Finite Cooling/Heating Rates: An Asymptotic Solution

**DOI:** 10.3390/ma4122108

**Published:** 2011-12-08

**Authors:** Zhihe Jin

**Affiliations:** Department of Mechanical Engineering, University of Maine, Orono, ME 04469, USA; E-Mail: zhihe.jin@maine.edu; Tel.: 1-207-581-2135; Fax: 1-207-581-2379

**Keywords:** functionally graded material, heat conduction, cooling rate, temperature, asymptotic solution

## Abstract

This work investigates transient heat conduction in a functionally graded plate (FGM plate) subjected to gradual cooling/heating at its boundaries. The thermal properties of the FGM are assumed to be continuous and piecewise differentiable functions of the coordinate in the plate thickness direction. A linear ramp function describes the cooling/heating rates at the plate boundaries. A multi-layered material model and Laplace transform are employed to obtain the transformed temperatures at the interfaces between the layers. An asymptotic analysis and an integration technique are then used to obtain a closed form asymptotic solution of the temperature field in the FGM plate for short times. The thermal stress intensity factor (TSIF) for an edge crack in the FGM plate calculated based on the asymptotic temperature solution shows that the asymptotic solution can capture the peak TSIFs under the finite cooling rate conditions.

## 1. Introduction

Functionally graded materials (FGMs) represent a new concept of tailoring materials with microstructural and properties gradients to achieve optimized performance. For high-performance structural applications, FGMs are often multi-phased composite materials with the volume fractions of their constituents varying gradually in pre-determined profiles. Thermal loads on FGM structures in high temperature applications induce severe thermal stresses which may lead to failure of the structural components. One of the original objectives of introducing FGMs is to employ the material property gradients to alter the temperature distribution, thereby possibly reducing thermal stresses in thermal structures. The optimal design and thermal stress analyses of FGMs to improve their high temperature and thermal fracture resistance often rely on the transient temperature solution for a long FGM plate with arbitrarily graded material properties in the thickness direction and analytical expressions of the temperature solution are desirable. The temperature field is typically one-dimensional (1-D) in the thickness direction in many structural applications. Obata and Noda [1] obtained a perturbation solution of 1-D heat conduction in an FGM plate. Ishiguro *et al.* [2] analyzed the 1-D temperature distribution in an FGM strip using a multi-layered material model. Tanigawa *et al.* [3] modeled an FGM plate by a laminated composite with homogeneous layers and obtained the solution of the 1-D temperature field. A layered material model was also used by Jin and Paulino [4] to obtain an approximate short time solution of temperature field in an FGM strip. In general, the studies based on the multi-layered model involved complicated series form solutions and the series converge very slowly at short times. On the other hand, the temperature solution at short times is particularly useful because thermal stresses and thermal stress intensity factors may reach their peak values in a very short period of time and these peak values govern the thermal stress failure of materials. Jin [5] obtained a simple closed form short time asymptotic solution of temperature field in an FGM strip with continuous and piecewise differentiable material properties under sudden cooling boundary conditions.

This paper extends the method in [5] to investigate 1-D heat conduction in an FGM plate with continuous and piecewise differentiable properties subjected to finite cooling/heating rates at the plate boundaries. The rates of temperature variation at the plate surfaces are described by a linear ramp function. A multi-layered material model is first used. The Laplace transform with its asymptotic properties and an integration technique are then employed to obtain a closed form, short time solution of temperature distribution. Finally, the thermal stress intensity factor for an edge crack in the FGM plate is calculated using the asymptotic temperature solution. 

## 2. Basic Equations

**Figure 1:**
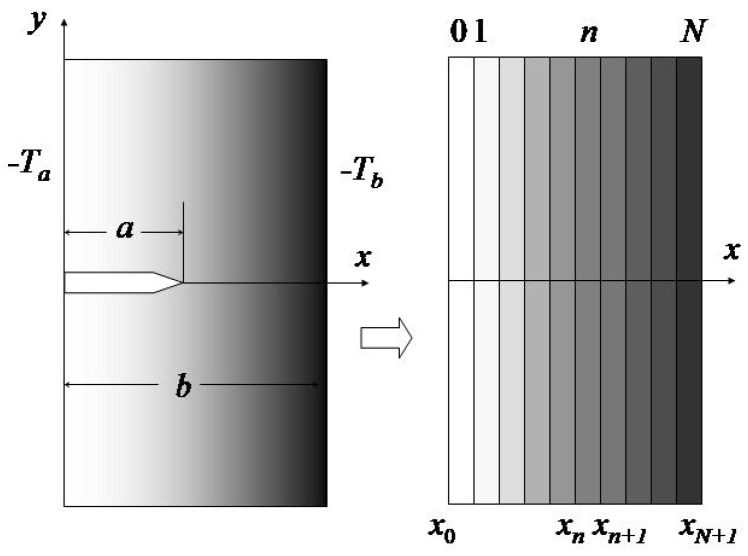
An FGM plate subjected to a thermal shock and a multi-layered material model.

Consider an FGM plate of thickness *b* as shown in Figure 1. The material properties of the FGM are graded in the thickness direction (*x –* direction). Initially the temperature of the plate is a constant *T*_0_ which can be assumed to be zero without loss of generality. The temperature then gradually changes to −*T*_a_ and −*T*_b_ at the surfaces *x* = 0 and *x = b* of the plate, respectively. We use a linear ramp function to describe the variations of the boundary temperatures. The initial and boundary conditions for the heat conduction problem are thus

(1) T(x,0)=0,      0≤x≤b

(2a) $$T(0,t)=\left\{ \begin{array}{l} -T_{a}\left(t/t_{a}\right)\text{, }0\leq t\leq t_{a}\\ -T_{a}\text{, }t>t_{a} \end{array}\right.$$

(2b) $$T(b,t)=\left\{ \begin{array}{l} -T_{b}\left(t/t_{b}\right)\text{, }0\leq t\leq t_{b}\\ \;-T_{b}\text{, }t>t_{b} \end{array}\right.$$

where *T* = *T*(*x*, *t*) is the temperature, *t* is time, and *t*_a_ and *t*_b_ are two temporal parameters describing the rates of temperature variation (cooling/heating rates) at the plate surfaces. The one-dimensional heat conduction in the plate is governed by the following basic equation

(3) $$\frac{\partial }{\partial x}\left[k(x)\frac{\partial T}{\partial x}\right]=\rho (x)c(x)\frac{\partial T}{\partial t}$$

where *k*(*x*) is the thermal conductivity, *ρ*(*x*) the mass density, and *c*(*x*) the specific heat. 

## 3. A Multi-Layered Material Model and the Discrete Temperature Solution

Following Reference [5], this work employs a multi-layered material model, the asymptotic property of Laplace transform and an integration technique to solve the heat conduction problem described by Equations (1–3). The plate is first divided into *N +* 1 layers in the thickness direction, as shown in Figure 1. The coordinates of the interfaces between the layers are denoted by *x*_n_ (*n* = 1, 2, …, *N*) and the two boundaries of the plate are *x*_0_ = 0 and *x*_N+1_ = *b*. When *N* becomes large, each layer may approximately be regarded as a homogeneous layer with constant properties *k*_n_ (thermal conductivity), *ρ*_n_ (density), *c*_n_ (specific heat), and *κ*_n_ = *k*_n_/(ρ_n_*c*_n_) (thermal diffusivity). Let *T*_n_ denote the temperatures at *x* = *x*_n_ (*n* = 0, 1, 2, …, *N*+1). The temperature within the *n*th layer under the initial condition, Equation (1), has the form [6,7]

(4) $$\begin{array}{ll} T(x*,\tau ) & =(1-x*)T_{n}(\tau )+x*T_{n+1}(\tau )\\ & -2\sum_{l=1}^{\infty }\frac{\sin(l\pi x*)}{l\pi }\left[T_{n}(\tau )-\beta_{nl}\int_{0}^{\tau }\exp\left(-\beta_{nl}(\tau -\tau^{\prime })\right)T_{n}(\tau^{\prime })d\tau^{\prime }\right]\\ & +2\sum_{l=1}^{\infty }{\left(-1\right)}^{l}\frac{\sin(l\pi x*)}{l\pi }\left[T_{n+1}(\tau )-\beta_{nl}\int_{0}^{\tau }\exp\left(-\beta_{nl}(\tau -\tau^{\prime })\right)T_{n+1}(\tau^{\prime })d\tau^{\prime }\right]\;\\ & 0<x*<1,\;n=0,\;1,\;\ldots ,\;N \end{array}$$

where x* and *τ* are the nondimensional local coordinate and time defined by

(5) $$x^{*}=(x-x_{n})/(x_{n+1}-x_{n}),\;\tau =t\kappa_{0}/b^{2}$$

respectively, and *β*_nl_ is a constant given by

(6) $$\beta_{nl}=\frac{\kappa_{n}}{\kappa_{0}}{\left(\frac{b}{h_{n}}\right)}^{2}{\left(l\pi \right)}^{2},\;h_{n}=x_{n+1}-x_{n}$$

The unknown interface temperatures *T*_n_(*τ*) (*n* = 1, 2, ..., *N*) are determined from the heat flux continuity conditions across the interfaces. 

(7) $$k_{n-1}{\frac{\partial T}{\partial x}|}_{x\to x_{n}^{-}}=k_{n}{\frac{\partial T}{\partial x}|}_{x\to x_{n}^{+}},\;n=1,2,\ldots ,N$$

Substitution of Equation (4) into Equation (7) yields a system of Volterra integral equations for *T*_n_(*τ*). The corresponding Laplace transformed equations have the form:

(8) $$\sum_{n=1}^{N}a_{mn}(s)\frac{{\bar{T}}_{n}(s)}{T_{a}}=b_{m}(s),\;m=1,\;2,\;\ldots ,\;N$$

where ${\bar{T}}_{n}(s)$ is the Laplace transform of *T*_n_(*τ*). In Equation (8), the nonzero coefficients *a*_mn_(*s*) have the same expressions as those for sudden cooling conditions (*t*_a_ → 0, *t*_b_ → 0) in Reference [5], and *b*_m_(*s*) have the forms:

(9) $$\begin{array}{l} b_{1}(s)=-\frac{k_{0}}{k_{1}}G_{0}(s)\frac{1-\exp\left(-s\tau_{a}\right)}{s^{2}\tau_{a}},\\ b_{m}(s)=0,\;m=2,3,\ldots ,N-1,\;\\ b_{N}(s)=-\left(\frac{T_{b}}{T_{a}}\right)\frac{h_{N-1}}{h_{N}}G_{N}(s)\frac{1-\exp\left(-s\tau_{b}\right)}{s^{2}\tau_{b}} \end{array}$$

where *τ*_a_ and *τ*_b_ are nondimensional temporal parameters defined by

(10) $$\tau_{a}=t_{a}\kappa_{0}/b^{2}\text{, }\tau_{b}=t_{b}\kappa_{0}/b^{2}$$

and *G*_n_(s) are given by

(11) $$G_{n}(s)=\frac{\sqrt{s}}{\gamma_{n}}\frac{1}{\sinh\left(\sqrt{s}/\gamma_{n}\right)},\;\gamma_{n}=\sqrt{\frac{\kappa_{n}}{\kappa_{0}}}\left(\frac{b}{h_{n}}\right)\text{, }n=0,1,\ldots ,N$$

Equation (8) generally does not permit closed form solutions. For large values of *s*, however, the Laplace transformed interface temperatures may be obtained as follows

(12) $$\begin{array}{ll} \frac{{\bar{T}}_{n}(s)}{T_{a}} & =\frac{L_{n}^{\left(0\right)}}{\tau_{a}}\frac{1-\exp(-s\tau_{a})}{s^{2}}\exp\left(-\sqrt{s}\sum_{i=1}^{n}\;\frac{1}{\gamma_{i-1}}\right)\\ & +\left(\frac{T_{b}}{T_{a}}\right)\frac{P_{n}^{\left(0\right)}}{\tau_{b}}\frac{1-\exp(-s\tau_{b})}{s^{2}}\exp\left(-\sqrt{s}\sum_{i=n}^{N}\;\frac{1}{\gamma_{i}}\right),\;n=1,2,\ldots ,N \end{array}$$

where $L_{n}^{\left(0\right)}$ and $P_{n}^{\left(0\right)}$ are the constants given in Reference [5]. By using the inverse Laplace transform, we can obtain the interface temperatures *T*_n_(τ) for short times as follows (*τ* << 1):

(13) $$\frac{T_{n}(\tau )}{T_{a}}=T_{n}^{\left(1\right)}(\tau )+\left(\frac{T_{b}}{T_{a}}\right)\;T_{n}^{\left(2\right)}(\tau )$$

where

(14) $$\begin{array}{l} T_{n}^{\left(1\right)}(\tau )=\frac{L_{n}^{\left(0\right)}}{\tau_{a}}\int_{0}^{\tau }erfc\left(\frac{1}{2\sqrt{\tau^{\prime }}}\sum_{i=1}^{n}\;\frac{1}{\gamma_{i}}\right)d\tau^{\prime },\;\tau \leq \tau_{a}\\ T_{n}^{\left(1\right)}(\tau )=\frac{L_{n}^{\left(0\right)}}{\tau_{a}}\int_{\tau -\tau_{a}}^{\tau }erfc\left(\frac{1}{2\sqrt{\tau^{\prime }}}\sum_{i=1}^{n}\;\frac{1}{\gamma_{i}}\right)d\tau^{\prime },\;\tau >\tau_{a} \end{array}$$

and

(15) $$\begin{array}{l} T_{n}^{\left(2\right)}(\tau )=\frac{P_{n}^{\left(0\right)}}{\tau_{b}}\int_{0}^{\tau }erfc\left(\frac{1}{2\sqrt{\tau^{\prime }}}\sum_{i=n}^{N}\;\frac{1}{\gamma_{i}}\right)d\tau^{\prime },\;\tau \leq \tau_{b}\\ T_{n}^{\left(2\right)}(\tau )=\frac{P_{n}^{\left(0\right)}}{\tau_{b}}\int_{\tau -\tau_{b}}^{\tau }erfc\left(\frac{1}{2\sqrt{\tau^{\prime }}}\sum_{i=n}^{N}\;\frac{1}{\gamma_{i}}\right)d\tau^{\prime },\;\tau >\tau_{b} \end{array}$$

where *erfc*( ) is the complementary error function. We note that the temporal parameters *τ*_a_ and *τ*_b_ should also be small, *i.e.*, the cooling/heating rates are finite but still relatively high.

## 4. A Closed Form Short Time Solution

In order to obtain a closed form temperature solution for an FGM with continuous and piecewise differential thermal properties from the interface temperatures Equations (13–15), we let the layer thicknesses go to zero and the number of layers go to infinite, *i.e.*, *x*_n+1_ – *x*_n_ → 0 (*n* = 0, 1, 2, …, *N*) and *N* → ∞. The limits of $L_{n}^{\left(0\right)}$ and $P_{n}^{\left(0\right)}$ in Equations (14) and (15) can be found as follows [5]:

(16a) $$L_{n}^{\left(0\right)}\to -{\left[\frac{\rho_{0}c_{0}k_{0}}{\rho (x_{n})c(x_{n})k(x_{n})}\right]}^{1/4},\;N\to \infty$$

(16b) $$P_{n}^{\left(0\right)}\to -{\left[\frac{\rho (b)c(b)k(b)}{\rho (x_{n})c(x_{n})k(x_{n})}\right]}^{1/4},\;N\to \infty$$

Substituting the equations above into Equations (13–15), we obtain a closed form solution of the temperature field for short times in the FGM plate with continuous and piecewise differentiable properties as follows:

(17) $$\frac{T(x,\tau )}{T_{a}}=T^{\left(1\right)}(x,\tau )+\left(\frac{T_{b}}{T_{a}}\right)\;T^{\left(2\right)}(x,\tau )$$

where $T^{\left(1\right)}(x,\tau )$ and $T^{\left(2\right)}(x,\tau )$ are given by

(18a) $$\begin{array}{ll} T^{\left(1\right)}(x,\tau ) & =-\frac{1}{\tau_{a}}{\left[\frac{\rho_{0}c_{0}k_{0}}{\rho (x)c(x)k(x)}\right]}^{1/4}\\ & \times \left\{\left[\tau +\frac{1}{2}\Omega_{1}^{2}(x)\right]erfc\left(\frac{\Omega_{1}(x)}{2\sqrt{\tau }}\right)-\frac{\Omega_{1}(x)}{\sqrt{\pi }}\sqrt{\tau }\exp\left[-\frac{\Omega_{1}^{2}(x)}{4\tau }\right]\right\},\;\tau \leq \tau_{a} \end{array}$$

(18b) $$\begin{array}{ll} T^{\left(1\right)}(x,\tau ) & =-\frac{1}{\tau_{a}}{\left[\frac{\rho_{0}c_{0}k_{0}}{\rho (x)c(x)k(x)}\right]}^{1/4}\{\left[\tau +\frac{1}{2}\Omega_{1}^{2}(x)\right]erfc\left(\frac{\Omega_{1}(x)}{2\sqrt{\tau }}\right)-\frac{\Omega_{1}(x)}{\sqrt{\pi }}\sqrt{\tau }\exp\left[-\frac{\Omega_{1}^{2}(x)}{4\tau }\right]\\ & -\left[\tau -\tau_{a}+\frac{1}{2}\Omega_{1}^{2}(x)\right]erfc\left(\frac{\Omega_{1}(x)}{2\sqrt{\tau -\tau_{a}}}\right)\;+\frac{\Omega_{1}(x)}{\sqrt{\pi }}\sqrt{\tau -\tau_{a}}\exp\left[-\frac{\Omega_{1}^{2}(x)}{4\left(\tau -\tau_{a}\right)}\right]\},\;\tau >\tau_{a} \end{array}$$

and

(19a) $$\begin{array}{ll} T^{\left(2\right)}(x,\tau ) & =-\frac{1}{\tau_{b}}{\left[\frac{\rho (b)c(b)k(b)}{\rho (x)c(x)k(x)}\right]}^{1/4}\\ & \times \left\{\left[\tau +\frac{1}{2}\Omega_{2}^{2}(x)\right]erfc\left(\frac{\Omega_{2}(x)}{2\sqrt{\tau }}\right)-\frac{\Omega_{2}(x)}{\sqrt{\pi }}\sqrt{\tau }\exp\left[-\frac{\Omega_{2}^{2}(x)}{4\tau }\right]\right\},\;\tau \leq \tau_{b}\; \end{array}$$

(19b) $$\begin{array}{ll} T^{\left(2\right)}(x,\tau ) & =-\frac{1}{\tau_{b}}{\left[\frac{\rho (b)c(b)k(b)}{\rho (x)c(x)k(x)}\right]}^{1/4}\{\left[\tau +\frac{1}{2}\Omega_{2}(x)\right]erfc\left(\frac{\Omega_{2}(x)}{2\sqrt{\tau }}\right)-\frac{\Omega_{2}(x)}{\sqrt{\pi }}\sqrt{\tau }\exp\left[-\frac{\Omega_{2}^{2}(x)}{4\tau }\right]\\ & -\left[\tau -\tau_{b}+\frac{1}{2}\Omega_{2}^{2}(x)\right]erfc\left(\frac{\Omega_{2}(x)}{2\sqrt{\tau -\tau_{b}}}\right)\;+\frac{\Omega_{2}(x)}{\sqrt{\pi }}\sqrt{\tau -\tau_{b}}\exp\left[-\frac{\Omega_{2}^{2}(x)}{4\left(\tau -\tau_{b}\right)}\right]\},\;\tau >\tau_{b} \end{array}$$

respectively. In Equations (18) and (19), Ω_1_(*x*) and Ω_2_(*x*) are defined by

(20) $$\begin{array}{l} \Omega_{1}(x)=\frac{1}{b}\int_{0}^{x}\sqrt{\frac{\kappa_{0}}{\kappa (x^{\prime })}}\;{dx}^{\prime },\\ \Omega_{2}(x)=\frac{1}{b}\int_{x}^{b}\sqrt{\frac{\kappa_{0}}{\kappa (x^{\prime })}}\;{dx}^{\prime } \end{array}$$

When *τ*_a_ and *τ*_b_ approach zero,$T^{\left(1\right)}(x,\tau )$ and $T^{\left(2\right)}(x,\tau )$ reduce to

(21a) $$T^{\left(1\right)}(x,\tau )=-{\left[\frac{\rho_{0}c_{0}k_{0}}{\rho (x)c(x)k(x)}\right]}^{1/4}erfc\left(\frac{\Omega_{1}(x)}{2\sqrt{\tau }}\right),\;\tau_{a}\to \text{0}$$

(21b) $$T^{\left(2\right)}(x,\tau )=-{\left[\frac{\rho (b)c(b)k(b)}{\rho (x_{n})c(x_{n})k(x_{n})}\right]}^{1/4}erfc\left(\frac{\Omega_{2}(x)}{2\sqrt{\tau }}\right),\;\tau_{b}\to \text{0}$$

which are the same as those under the sudden cooling conditions [5].

Equations (17–20) represent an asymptotic solution of temperature for short times. To gain an understanding of the applicability region in which the asymptotic solution is valid, the asymptotic solution is applied to a homogeneous plate with *T*_b_ = 0 in the boundary condition Equation (2b) by taking *k*(*x*) = *k*_0_, *c*(*x*) = *c*_0_, *ρ*(*x*) = *ρ*_0_ and *κ*(*x*) = *κ*_0_ in Equations (17–20). The complete solution of temperature in this case is

(22) $$\begin{array}{l} \frac{T(x,\tau )}{T_{a}}=-\left(1-\frac{x}{b}\right)\frac{\tau }{\tau_{a}}\;+\frac{2}{\tau_{a}}\sum_{m=1}^{\infty }\frac{\sin(m\pi x/b)}{m^{3}\pi^{3}}\left[1-\exp\left(-m^{2}\pi^{2}\tau \right)\right],\;0<\tau \leq \tau_{a,}\\ \frac{T(x,\tau )}{T_{a}}=-\left(1-\frac{x}{b}\right)\;+\frac{2}{\tau_{a}}\sum_{m=1}^{\infty }\frac{\sin(m\pi x/b)}{m^{3}\pi^{3}}\left[\exp\left(-m^{2}\pi^{2}(\tau -\tau_{a})\right)-\exp\left(-m^{2}\pi^{2}\tau \right)\right],\;\tau >\tau_{a} \end{array}$$

**Figure 2:**
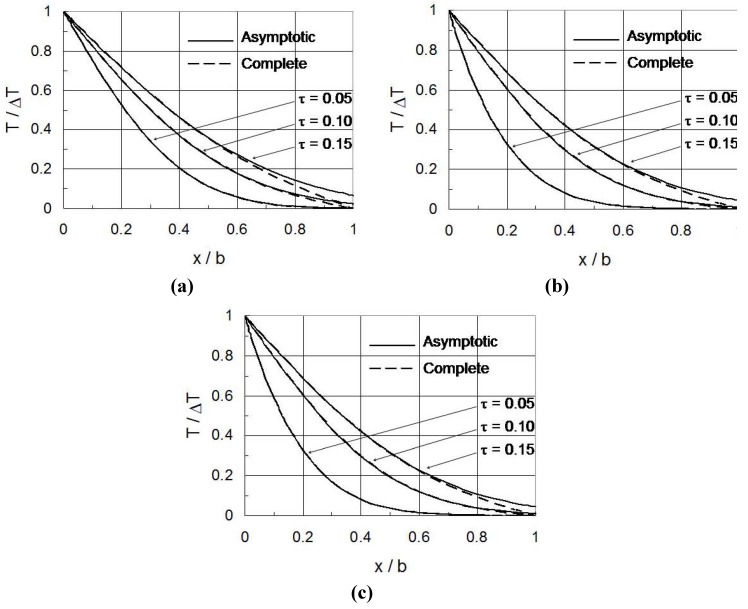
Temperature field: asymptotic and complete solutions for a homogeneous plate. (**a**) *τ*_a_ = 0.001; (**b**) *τ*_a_ = 0.05; (**c**) *τ*_a_ = 0.1.

Figure 2a shows the normalized temperature (where Δ*T* = − *T*_a_) *versus* nondimensional coordinate (*x*/*b*) at various nondimensional time *τ*. The temporal parameter *τ*_a_ is 0.001. The asymptotic solution almost coincides with the complete solution in the entire plate for nondimensional times up to *τ* = 0.05. Those solutions are also in good agreement in the region of *x*/*b* < 0.8 for times up to *τ* = 0.10. For times up to *τ* = 0.15, the solutions agree well with each other in the region of *x*/*b* < 0.6. The asymptotic solution approximately satisfies the boundary condition at *x* = *b* for *τ* < 0.05. Figure 2b and Figure 2c show similar results when the parameter *τ*_a_ is increased to 0.05 and 0.1, respectively. It appears that *τ*_a_ or the rate of boundary temperature variation does not significantly influence the applicability region of the short time solution. It is expected that the short time solution for an FGM plate will also be approximately valid for nondimensional times up to *τ* = 0.10 if the material property gradation is not extremely steep. 

## 5. Effects of Cooling Rate on the Thermal Stress Intensity Factor for an Edge Crack in an FGM Plate

This section uses the asymptotic temperature solution Equations (17–20) to calculate the thermal stress intensity factor (TSIF) for an edge crack in an elastically homogeneous but thermally graded FGM plate (see Figure 1) and demonstrates that the short time temperature solution can capture the peak TSIF. The integral equation method is employed and the singular integral equation of the crack problem is given as follows:

(23) $$\int_{\text{-1}}^{\text{1}}\left[\frac{1}{s-r}+K(r,s)\right]\;f\text{(}s\text{)}ds=-\frac{2\pi (1-\nu^{2})}{E}\sigma_{yy}^{T}(r,\tau ),\;|r|\leq 1$$

where *f*(*x*) is the basic unknown function defined by

(24) $$f(x)=\frac{\partial v(x,0)}{\partial x}$$

with v(*x*,0) being the crack surface displacement in the *y* direction , *K*(*r, s*) is a known kernel [4], *r* and *s* are the nondimensional coordinates given by

(25) $$r=2x/a-1,\;s=2x^{\prime }/a-1$$

and $\sigma_{yy}^{T}$ is the thermal stress given by

(26) $$\sigma_{yy}^{T}(x,t)=-\frac{E\alpha \theta (x,t)}{1-\nu }+\frac{E}{1-\nu }[\frac{4b-6x}{b^{2}}\int_{0}^{b}\alpha \theta (x,t)dx-\frac{6b-12x}{b^{3}}\int_{0}^{b}x\alpha \theta (x,t)dx]$$

in which *E* is Young’s modulus, ν Poisson’s ratio, *α* = *α*(*x*) the coefficient of thermal expansion, and *θ*(*x*, *t*) = *T*(*x*, *t*) – *T*_0_ the temperature variation.

According to the singular integral equation theory [8], the solution of Equaion (23) has the following form

(27) $$f(r)=\frac{F(r)}{\sqrt{1-r}}$$

where *F*(*r*) is a continuous and bounded function. Once the solution of Equation (23) is obtained, the TSIF at the crack tip can be computed from

(28) $$K^{*}=\frac{(1-\nu )K_{I}}{E\alpha_{0}T_{a}\sqrt{\pi \;b}}\;=-\frac{1}{2}\sqrt{\frac{a}{b}}\;F\text{(}1\text{)}$$

where *K*_I_ denotes the TSIF, K^*^ the nondimensional TSIF, and α_0_ = α(0). In Equation (28) *F*(1) is a function of time *τ*.

We use a TiC/SiC graded system to examine the effects of cooling rate on the TSIF. The FGM is a two-phase composite material with graded volume fractions of its constituent phases. The volume fraction of SiC is assumed to follow a simple power function

(29) $$V(x)={\left(x/b\right)}^{p}$$

where *p* is the exponent determining the volume fraction profile. The material properties of the FGM are calculated using conventional micromechanics models [4] and the properties of TiC and SiC are given in Table 1.

**Table 1:** Material properties of TiC and SiC.

Materials	Young’s modulus (GPa)	Poisson’s ratio	CTE (10^−6^/K)	Thermal conductivity (W/m-K)	Mass density (g/cm^3^)	Specific heat (J/g-K)
TiC	400	0.2	7.0	20	4.9	0.7
SiC	400	0.2	4.0	60	3.2	1.0

**Figure 3:**
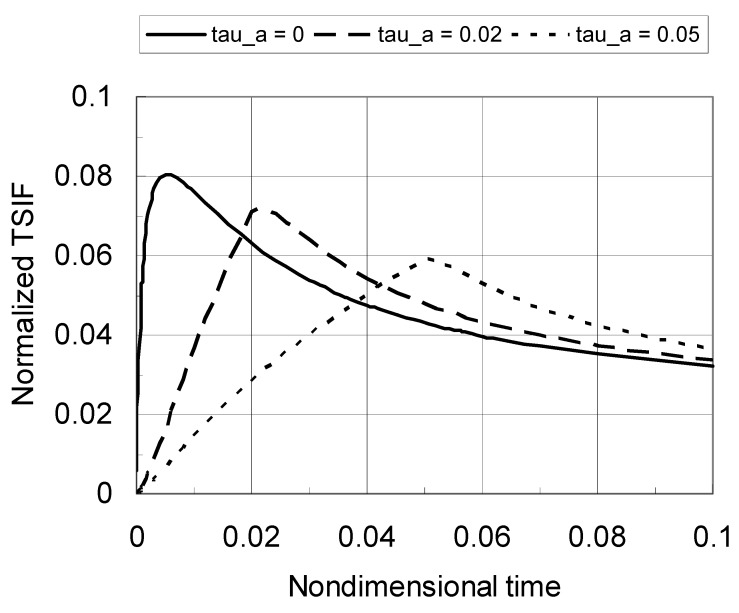
Normalized TSIF *versus* nondimensional time for a TiC/SiC FGM (*a*/*b* = 0.1, *p* = 0.2).

Figure 3 shows the normalized TSIF *versus* nondimensional time for various values of the cooling rate parameter *τ*_a_. The crack length is *a*/*b* = 0.1 and the material gradation profile index is *p* = 0.2. The TSIF under the sudden cooling condition (*τ*_a_ = 0, and hence infinite cooling rate) is also included. For a given cooling rate (*T*_a_/*τ*_a_), the TSIF initially increases with time, rapidly reaches the peak value and then decreases with time. The peak TSIF decreases significantly with a decrease in the cooling rate (increasing *τ*_a_). Moreover, the time at which the TSIF reaches its peak increases with a decrease in the cooling rate. 

**Figure 4:**
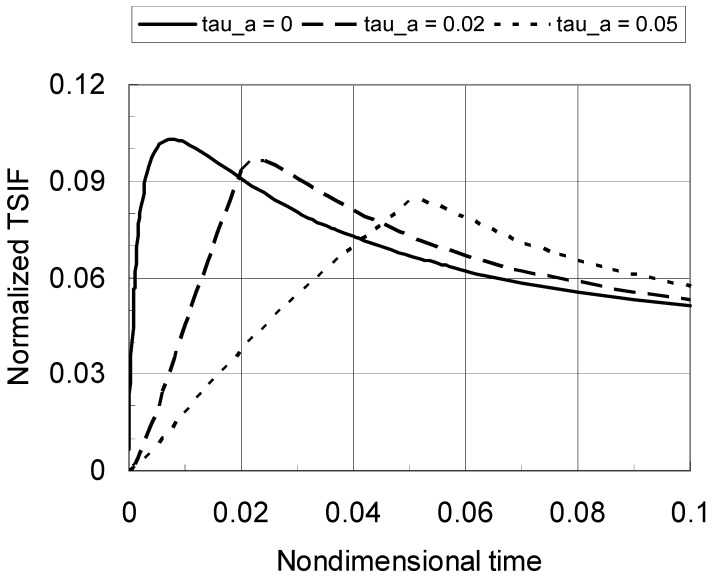
Normalized TSIF *versus* nondimensional time for a TiC/SiC FGM (*a*/*b* = 0.1, *p* = 0.5).

Figure 4 shows the normalized TSIF *versus* nondimensional time with an increased material gradation profile index of *p* = 0.5. The crack length is still *a*/*b* = 0.1. Again, the peak TSIF decrease significantly with a decrease in the cooling rate. Comparing the results in Figure 3 and Figure 4, one can find that the peak TSIF is reduced by a decrease in the material gradation index *p*. Clearly, the peak TSIF occurs at nondimensional times less than 0.1, which indicates that the asymptotic temperature solution can be used to capture the peak TSIF.

## 5. Concluding Remarks

A closed form, short time solution of heat conduction in an FGM plate with continuous and piecewise differentiable material properties is obtained using a multi-layered material model, the asymptotic property of Laplace transform, and an integration technique. The rates of temperature variations at the surfaces of the plate are taken into account and are described by a linear ramp function. Application of the asymptotic solution to a homogeneous plate indicates that the asymptotic solution agrees well with the complete solution for nondimensional times up to about *τ* = 0.10. It is found that the rates of temperature variation at the plate surfaces have an insignificant effect on the applicability region of the short time solution. The significance of the solution lies in the fact that the TSIF induced by the thermal shock reaches its peak value in a very short period of time as seen in Section 4. Thus, the solution may be used to evaluate the peak values of TSIF and the critical thermal shocks that cause crack propagation.

## References

[1] Obata Y., Noda N. (1993). Unsteady thermal stresses in a functionally gradient material plate. Trans. Jpn. Soc. Mech. Eng. Ser. A.

[2] Ishiguro T., Makino A., Araki N., Noda N. (1993). Transient temperature response in functionally gradient materials. Int. J. Thermophys..

[3] Tanigawa Y., Akai T., Kawamura R., Oka N. (1996). Transient heat conduction and thermal stress problems ofa nonhomogeneous plate with temperature-dependent material properties. J. Therm. Stress..

[4] Jin Z.-H., Paulino G.H. (2001). Transient thermal stress analysis of an edge crack in a functionally graded material. Int. J. Fract..

[5] Jin Z.-H. (2002). An asymptotic solution of temperature field in a strip of a functionally graded material. Int. Commun. Heat Mass Transf..

[6] Carslaw H.S., Jaeger J.C. (1959). Conduction of Heat in Solids.

[7] Ozisik M.N. (1980). Heat Conduction.

[8] Kaya A.C., Erdogan F. (1987). On the solution of integral equations with strongly singular kernels. Q. Appl. Math..

